# Retinal and Choroidal Microvascular Alterations Associated with Compensatory Head Tilt in Congenital Superior Oblique Palsy: An Interocular OCTA Analysis

**DOI:** 10.3390/jcm15134906

**Published:** 2026-06-24

**Authors:** Osman Parca, Tunahan Akyol, Emine Seker Un, Beyzanur Yıldız

**Affiliations:** 1 Department of Ophthalmology, Faculty of Medicine, Pamukkale University, Pamukkale 20160, Denizli, Turkey; osmanparca@gmail.com (O.P.); beyzayildiz1205@gmail.com (B.Y.); 2 Department of Ophthalmology, Egekent Hospital, Denizli 20125, Turkey; urositki@gmail.com

**Keywords:** congenital superior oblique palsy, head tilt, optical coherence tomography angiography, choroidal vascularity index, retinal microcirculation, choroid

## Abstract

**Background/Objectives**: To evaluate interocular retinal and choroidal microvascular alterations associated with compensatory head tilt in unilateral congenital superior oblique palsy (SOP) and to investigate their relationship with head tilt degree using optical coherence tomography angiography (OCTA). **Methods**: This retrospective cross-sectional study included 34 patients with congenital SOP and head tilt–dominant abnormal head posture. A paired-eye design compared the tilt-side eye with the opposite-side eye. Superficial and deep capillary plexus (SCP, DCP) vessel density, foveal avascular zone (FAZ) parameters, choroidal vascularity index (CVI), and subfoveal choroidal thickness (SFCT) were assessed. Interocular differences were defined as Δ = opposite-side eye − tilt-side eye. Correlation and multivariable regression analyses were performed to assess associations with head tilt degree. The interocular difference in CVI (ΔCVI) and its association with head tilt degree were defined as the primary outcomes, whereas retinal OCTA parameters, FAZ metrics, and SFCT were considered exploratory secondary outcomes. **Results**: CVI showed the most prominent interocular difference, being higher in the opposite-side eye than in the tilt-side eye (0.71 ± 0.04 vs. 0.68 ± 0.04; *p* < 0.001), whereas SFCT did not differ significantly (*p* = 0.395). SCP foveal vessel density and DCP inferior vessel density showed nominal differences in the unadjusted analyses but did not remain statistically significant after Benjamini–Hochberg false discovery rate correction. ΔCVI correlated positively with head tilt degree (ρ = +0.533, *p* = 0.001) and remained independently associated in multivariable analysis (*p* = 0.001). **Conclusions**: Compensatory head tilt in congenital SOP is associated with measurable interocular microvascular asymmetry, predominantly at the choroidal level. CVI demonstrated the strongest association with head tilt severity, whereas retinal OCTA findings were exploratory, suggesting that OCTA may provide objective insight into posture-related choroidal microvascular alterations.

## 1. Introduction

Congenital superior oblique palsy (SOP) is one of the most common causes of cyclovertical strabismus in childhood and is characteristically associated with a compensatory abnormal head tilt. In these patients, the head tilt typically tilts toward the side opposite the affected eye to compensate for ocular misalignment, maintain single binocular vision, and optimize functional vision. This chronic postural adaptation, which begins in early childhood, is well known to be associated with permanent facial asymmetry, morphological changes related to hemifacial hypoplasia, and secondary musculoskeletal effects over time [1,2,3].

Long-standing abnormal head posture has been suggested to influence not only craniofacial morphology but also vascular hemodynamics. Experimental studies have demonstrated that changes in head position can induce measurable alterations in carotid artery geometry, flow patterns, and wall shear stress [3]. More recent clinical evidence further suggests that prolonged abnormal head posture in congenital fourth nerve palsy may be associated with asymmetric changes in carotid artery diameter and flow parameters [4]. These macrovascular observations provide a biological basis for the hypothesis that chronic compensatory head tilt may also affect retinal and choroidal microcirculation.

Optical coherence tomography angiography (OCTA) is a non-invasive imaging modality that enables rapid, quantitative evaluation of the retinal and choroidal microvascular networks [5]. Previous studies have demonstrated that measurable changes in retinal and choroidal microcirculation can occur in association with strabismus and following strabismus surgery [6,7]. More recently, alterations in retinal vascular density have been reported in congenital unilateral trochlear nerve palsy, indicating that microvascular effects in this patient group are both detectable and quantifiable [8]. The choroidal vascularity index (CVI) has also gained importance as a biomarker for assessing choroidal vascular status, providing complementary information to choroidal thickness measurements [9]. In this context, CVI appears particularly valuable for evaluating the potential impact of chronic postural exposure on the choroid.

Accordingly, in the present study, we aimed to evaluate interocular retinal and choroidal microvascular changes associated with chronic head tilt in patients with unilateral congenital SOP and pronounced compensatory head tilt, and to investigate the relationship between these changes and the degree of head tilt using OCTA-based imaging.

## 2. Materials and Methods

This retrospective cross-sectional study was conducted in accordance with the principles of the Declaration of Helsinki following approval from the local ethics committee (Date: 2 December 2025; Approval No: E-60116787-020-796767). Patient records from January 2022 to November 2025 were reviewed retrospectively. Patients with a confirmed diagnosis of congenital SOP based on the Parks–Bielschowsky three-step test and presenting with compensatory abnormal head posture were included in the study [10]. All cases were evaluated at a tertiary eye care center.

The inclusion criteria were as follows: a clinically confirmed diagnosis of congenital SOP, a prominent head-tilt component in the habitual head position, and availability of high-quality OCTA and structural optical coherence tomography images. To minimize potential confounding effects, patients with a chin elevation or depression (pitch component) of ≥5° or with significant head rotation (yaw component) on clinical assessment were excluded. Additional exclusion criteria included a history of intraocular surgery, coexisting ocular pathology affecting the retina or choroid, glaucoma, high refractive error, anisometropia with a spherical equivalent difference of ≥1.5 diopters, systemic or ocular conditions that could affect microcirculation, and image artifacts compromising image quality. Written informed consent was obtained from all participants or their legal guardians.

The primary quantitative component of abnormal head posture, the head tilt angle, was measured from standardized frontal facial photographs obtained under uniform lighting conditions at a fixed distance using a tripod, while patients maintained their habitual head position and fixated on a distant target at eye level. The head tilt angle was defined as the angle between the interpupillary line and the true horizontal axis and was calculated in degrees using digital image analysis software [1,11]. Each measurement was independently performed by two observers on the same image, and the mean of the two measurements was used for analysis. Although the conventional classification of paretic and fellow eyes is commonly used in congenital SOP, compensatory head tilt typically occurs toward the side opposite the paretic eye, which may create directional ambiguity in studies focused on head tilt–related effects. Therefore, the present study classified eyes according to their relationship with the direction of compensatory head tilt: the eye on the side toward which the head was tilted was defined as the “tilt-side eye,” and the contralateral eye was defined as the “opposite-side eye.”

Retinal and choroidal imaging were performed during the same session using the Optos SOLIX device (Optos PLC, Dunfermline, UK). For retinal microvascular analysis, macula-centered 6 × 6 mm OCTA scans were obtained. All imaging procedures were conducted by the same experienced operator in accordance with a standardized protocol. Vessel density measurements were recorded for the superficial capillary plexus (SCP) and deep capillary plexus (DCP) in the whole image, foveal, parafoveal, superior, inferior, temporal, and nasal sectors. In addition, the boundaries of the foveal avascular zone (FAZ) were delineated within the SCP layer, and FAZ area (mm^2^), FAZ perimeter (mm), and FAZ circularity index were measured separately for each eye. Only images with sufficient quality and without segmentation errors were included in the analyses.

Choroidal evaluation was performed using horizontal structural OCT B-scan images acquired with the same device and passing through the fovea. The choroid was defined as the tissue extending from the retinal pigment epithelium to the choroid–sclera junction. Images were converted to grayscale and exported to ImageJ software (NIH, Bethesda, MD, USA) [12]. Binarization was then applied using the Otsu thresholding method within a manually selected region of interest centered on the fovea, spanning a 1500 µm horizontal section [13].

From this process, the total choroidal area, luminal area, and stromal area were calculated. The CVI was defined as the ratio of luminal area to total choroidal area and was used as the primary choroidal parameter in this study9. Subfoveal choroidal thickness (SFCT) was also recorded. CVI measurements were performed independently by two investigators, and the mean values were used for analysis. To minimize observer bias, the two independent investigators performing the ImageJ binarization and CVI measurements were masked to the eye classification (tilt-side eye versus opposite-side eye) during image analysis. All imaging procedures were conducted in accordance with the unit’s standardized imaging protocol under similar environmental conditions.

Data distribution was assessed using the Shapiro–Wilk test. Descriptive data were presented as mean ± standard deviation or median (interquartile range), as appropriate. Comparisons between the tilt-side eye and the opposite-side eye were performed using the paired samples t-test or the Wilcoxon signed-rank test, depending on data distribution.

Interocular differences were defined for all parameters as Δ = opposite-side eye − tilt-side eye. The primary outcome of the study was defined as the interocular difference in choroidal vascularity index (ΔCVI) and its association with head tilt degree. Retinal OCTA parameters, FAZ metrics, and subfoveal choroidal thickness were considered exploratory secondary outcomes. To account for multiple comparisons among exploratory secondary outcomes, Benjamini–Hochberg false discovery rate correction was applied. Associations between Δ values and head tilt angle, as well as age, were evaluated using Spearman correlation analysis. To assess the independent effect of head tilt angle on interocular choroidal asymmetry, a multivariable linear regression analysis was performed. Because of the interocular paired-eye study design, each participant served as their own control, thereby reducing the influence of subject-level confounders such as sex and systemic characteristics. Axial length demonstrated no significant interocular difference, and patients with anisometropia (spherical equivalent difference ≥1.5 diopters) were excluded. In addition, eyes were classified according to the direction of compensatory head tilt rather than anatomical laterality; therefore, right-versus-left laterality was not considered an independent confounding variable in the regression analysis.

Sample size adequacy was calculated using G*Power software for a paired-measurement design, assuming a medium effect size (Cohen’s dz = 0.55), 80% statistical power, and a 5% type I error rate, which indicated that a minimum of 28 patients was required. Statistical significance was set at *p* < 0.05. All analyses were performed using SPSS software (version 23; IBM Corp., Armonk, NY, USA).

## 3. Results

A total of 46 patients with congenital superior oblique palsy were initially screened for eligibility. Twelve patients were excluded because they presented with a pitch and/or yaw component of ≥5°, resulting in a final study cohort of 34 patients with head tilt–dominant abnormal head posture. The demographic and clinical characteristics of the study population are presented in Table 1.

Comparisons of ocular parameters between the tilt-side eye and the opposite-side eye are summarized in Table 2. Consistent with the paired-eye study design, axial length was similar between the two eyes (*p* = 0.830).

The most prominent interocular difference was observed in the CVI. The CVI was significantly higher in the opposite-side eye compared with the tilt-side eye (0.71 ± 0.04 vs. 0.68 ± 0.04; *p* < 0.001), and this parameter demonstrated the largest effect size among paired comparisons (Cohen’s dz = +0.735). In contrast, no significant difference was found between the two eyes in terms of SFCT (337.12 ± 58.47 µm vs. 343.68 ± 59.44 µm; *p* = 0.395).

No significant interocular asymmetry was observed for FAZ area, FAZ perimeter, or FAZ circularity index. Regarding exploratory retinal microvascular parameters, SCP foveal vessel density and DCP inferior sector vessel density showed nominal differences in the unadjusted analyses (*p* = 0.041 and *p* = 0.009, respectively). However, after Benjamini–Hochberg false discovery rate correction, SCP foveal vessel density (BH-adjusted *p* = 0.369) and DCP inferior vessel density (BH-adjusted *p* = 0.162) did not remain statistically significant. No significant interocular differences were found in the remaining SCP and DCP parameters.

**Figure 1:**
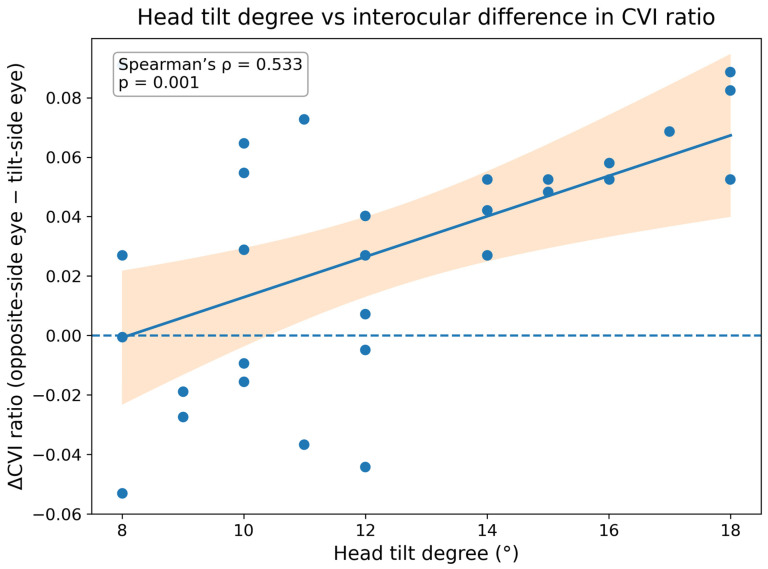
Scatter plot demonstrating the association between head tilt degree and the interocular difference in choroidal vascularity index (ΔCVI; opposite-side eye − tilt-side eye). The solid line represents the fitted regression line, and the shaded area indicates the corresponding 95% confidence interval. A significant positive correlation was observed between head tilt degree and ΔCVI (Spearman’s ρ = 0.533, *p* = 0.001).

Correlation analyses between interocular differences (Δ = opposite-side eye − tilt-side eye) and head tilt angle, as well as age, are presented in Table 3. The only parameter significantly associated with head tilt was ΔCVI. A statistically significant, moderate positive correlation was found between ΔCVI and head tilt angle (Spearman ρ = +0.533, *p* = 0.001), indicating that interocular differences in CVI became more pronounced with increasing head tilt severity (Figure 1).

The relationship between interocular differences in SFCT and head tilt angle did not reach statistical significance (ρ = +0.302, *p* = 0.083). Parameters showing significant associations with age were limited to the interocular difference in FAZ area (ρ = +0.356, *p* = 0.039) and parafoveal vessel density in the DCP (ρ = −0.376, *p* = 0.028). In contrast, no significant association was observed between age and ΔCVI (ρ = −0.046, *p* = 0.795). No significant correlations were found between head tilt angle or age and the remaining interocular difference parameters.

In the secondary multivariable linear regression analysis, the degree of head tilt was independently associated with the interocular difference in CVI (ΔCVI = opposite-side eye − tilt-side eye), whereas age was not. An increase in head tilt angle was associated with a higher positive ΔCVI (B = 0.0068, 95% CI: 0.003–0.011, *p* = 0.001). In contrast, no independent association was observed between age and ΔCVI (B = −0.0001, 95% CI: −0.002 to 0.002, *p* = 0.888). The model explained 30.9% of the variance in ΔCVI (R^2^ = 0.309).

## 4. Discussion

The principal finding of this study is that compensatory head tilt in unilateral congenital superior oblique palsy appears to be associated with a measurable interocular asymmetry predominantly at the choroidal level. In particular, the marked difference observed in CVI and its increase with greater head tilt severity suggest that the effects of head tilt on choroidal vascular composition may be reflected more sensitively by this parameter.

In contrast, the absence of a significant difference in SFCT between the two eyes indicates that the observed effect may be related more to alterations in the distribution of vascular components rather than to gross structural thickness changes. In addition, because retinal OCTA findings did not remain statistically significant after Benjamini–Hochberg false discovery rate correction, the present data support a more robust choroidal rather than retinal microvascular association with compensatory head tilt.

The CVI has gained prominence as a biomarker for evaluating choroidal vascular status, providing additional information beyond thickness measurements alone [9]. While choroidal thickness reflects the overall tissue volume, CVI offers a more functional perspective on the relative distribution of the vascular component within the choroid; therefore, it is considered a complementary parameter for detecting more subtle structural changes [9,14].

Indeed, studies conducted after strabismus surgery have reported measurable changes in choroidal microvascular structures, CVI, and choroidal thickness, suggesting that mechanical or hemodynamic alterations in the oculomotor system may have downstream effects on the choroid [6,7,15]. In this context, the finding in our study that SFCT remained similar between the two eyes, while CVI demonstrated significant interocular asymmetry, suggests that the effect associated with compensatory head tilt may be more closely related to changes in the relative distribution of choroidal vascular components than to gross structural thickness alterations. This distinction further supports the notion that CVI may be a more sensitive marker than SFCT for evaluating ocular effects associated with head tilt.

The vascular effects of head tilt may be considered not only at the ocular level but also in relation to more proximal hemodynamic structures. Experimental studies have shown that changes in head position can induce measurable alterations in carotid artery geometry, flow patterns, and wall shear stress [3]. Similarly, prolonged abnormal head posture in congenital fourth nerve palsy has been reported to be associated with asymmetric changes in carotid artery diameter and flow parameters [4]. In contrast, prospective observational data in pediatric strabismus patients did not demonstrate significant acute changes in common carotid blood flow during induced head tilt, although longer-term effects of abnormal head posture could not be excluded [16]. Therefore, current macrovascular evidence remains limited and should be interpreted cautiously.

These macrovascular observations provide a biological basis for the notion that compensatory head tilt may exert secondary effects on ocular circulation [17]. In our study, the finding that interocular differences in CVI increased in parallel with head-tilt severity suggests that these hemodynamic influences may also be reflected at the level of the choroidal vasculature.

Although SCP foveal vessel density and DCP inferior vessel density showed nominal differences in the unadjusted analyses, these findings did not remain statistically significant after Benjamini–Hochberg false discovery rate correction. Therefore, retinal OCTA findings should be regarded as exploratory, and no definitive conclusion can be drawn regarding retinal microvascular involvement in the present cohort.

In a recent OCTA study of congenital unilateral trochlear nerve palsy, central SCP and DCP vessel densities in the fellow eye were reported to be lower than those in both the paretic eye and healthy controls, and certain retinal vascular parameters showed an inverse relationship with the angle of deviation [8]. While these findings indicate that retinal microvascular alterations have been described in this patient group, our FDR-adjusted results suggest that the retinal findings in the present cohort should be interpreted cautiously and primarily as exploratory observations.

The positive association between head tilt angle and ΔCVI, demonstrated in both correlation analysis and the multivariable model, suggests that choroidal asymmetry may be quantitatively linked to postural severity. This indicates that interocular differences in CVI do not represent a fixed disparity between the two eyes but rather follow a pattern that becomes more pronounced as head tilt increases.

The persistence of this relationship after adjustment for age further supports the notion that the observed choroidal asymmetry is more closely related to the severity of head tilt. Although age was not independently associated with interocular CVI asymmetry in the present study, developmental maturation of the choroid during early childhood may influence baseline choroidal characteristics. Accordingly, the inclusion of younger participants may have introduced physiological variability in absolute choroidal parameters. Future studies with larger age-stratified cohorts are warranted to clarify the influence of developmental changes on posture-related microvascular alterations.

In the existing literature, compensatory head posture in congenital superior oblique palsy has primarily been evaluated in terms of facial asymmetry, musculoskeletal effects, and, more recently, retinal microvascular changes [1,2,8]. Our study extends this framework by simultaneously examining retinal and choroidal vascular parameters in patients with head-tilt–dominant unilateral SOP using an interocular comparative design.

Comparing the tilt-side eye with the opposite-side eye within the same individual reduces the influence of interindividual anatomical and systemic variability, while quantitatively including head tilt angle enables assessment of the relationship between vascular asymmetry and postural severity. In this context, our findings suggest that compensatory head tilt may represent not only a visible motor adaptation but also measurable alterations in ocular vasculature, with a more pronounced effect on the choroidal component.

The identification of interocular asymmetry in CVI and selected retinal OCTA parameters suggests that OCTA-based imaging may provide complementary objective information for monitoring posture-related microvascular alterations. However, its potential role in guiding surgical timing remains hypothetical and requires validation in prospective longitudinal studies. The potential clinical utility of OCTA-derived vascular parameters should currently be considered exploratory. Although our findings suggest that interocular CVI asymmetry reflects the severity of compensatory head tilt, whether these alterations are reversible after surgical correction of ocular misalignment or abnormal head posture remains unknown. Previous studies have demonstrated postoperative changes in choroidal vascular parameters following strabismus surgery, suggesting that at least some ocular vascular alterations may be dynamic rather than permanent [6,7]. Therefore, OCTA-derived biomarkers may eventually provide objective information for monitoring posture-related microvascular changes and postoperative recovery; however, prospective longitudinal studies are required before such parameters can be incorporated into clinical decision-making or surgical timing strategies.

The interpretation of our findings should be considered in light of the study design. The interocular comparative approach, focusing on head-tilt–dominant unilateral congenital superior oblique palsy, enabled a more targeted evaluation by minimizing the influence of interindividual anatomical and systemic variability. Quantitative inclusion of head tilt angle enabled assessment of the relationship between vascular asymmetry and postural severity, while simultaneous evaluation of retinal and choroidal parameters allowed interpretation of the observed effects across different circulatory components within a unified framework.

However, several limitations should be acknowledged. The retrospective, single-center design and relatively limited sample size may restrict the generalizability of the findings. In addition, although abnormal head posture is a three-dimensional clinical phenomenon, the present study focused specifically on the head-tilt component, excluding pitch and yaw. While this approach improved phenotypic homogeneity, it may limit the applicability of the results to other patterns of abnormal head posture. Although carotid and systemic hemodynamic alterations may provide a plausible biological explanation for the observed interocular vascular asymmetry, these mechanisms were not directly evaluated in the present study. Moreover, OCTA-derived parameters represent surrogate structural and vascular biomarkers rather than direct measurements of ocular blood flow; therefore, the observed associations should be interpreted with appropriate caution. Future studies incorporating direct hemodynamic assessments are warranted to validate the proposed biological pathways. Finally, the use of age as a surrogate marker for the duration of exposure should also be taken into consideration.

## 5. Conclusions

Compensatory head tilt in unilateral congenital superior oblique palsy appears to be associated with a measurable interocular asymmetry, particularly at the level of the choroidal vasculature. The marked difference observed in CVI and its increase with greater head-tilt severity highlights its potential as a sensitive indicator of the impact of postural adaptation on choroidal vascular composition.

In contrast, retinal OCTA findings did not remain statistically significant after correction for multiple comparisons and should therefore be regarded as exploratory. These findings indicate that compensatory head tilt may represent not only a motor adaptation but also a biological condition associated with measurable alterations in choroidal vascular organization.

Further studies with larger sample sizes, prospective designs, and multidimensional assessments of head posture are warranted to better elucidate the clinical significance and underlying mechanisms of this relationship.

## Figures and Tables

**Table 1:** Demographic and Clinical Characteristics of the Study Population.

Variable	Value
**Age (years)**	
Median (IQR)	11.0 (9.0–13.8)
Mean ± SD	13.0 ± 6.4
Range	4–27
**Sex, n (%)**	
Male	18 (52.9%)
Female	16 (47.1%)
**Head Tilt Direction, n (%)**	
Right	16 (47.1%)
Left	18 (52.9%)
**Head Tilt Degree (°)**	
Median (IQR)	12.0° (10.0–15.5°)
Mean ± SD	12.3 ± 3.4°
Range	8–18°

IQR, interquartile range; SD, standard deviation.

**Table 2:** Comparison of Ocular Parameters Between Head Tilt Side and Opposite Side Eyes.

Parameter	Head Tilt Side	Opposite Side	*p* Value	BH-Adjusted *p* ^†^	Cohen’s dz
Biometric Parameters					
Axial Length (mm)	23.2 ± 0.8	23.2 ± 0.9	0.830		+0.000
CVI and Choroid Parameters					
CVI Ratio	0.68 ± 0.04	0.71 ± 0.04	<0.001		+0.735
SFCT (µm)	343.68 ± 59.44	337.12 ± 58.47	0.395	0.910	−0.148
SCP Parameters					
SCP Whole VD (%)	49.85 (49.00–50.20)	49.80 (48.20–50.60)	0.629	0.910	+0.002
SCP Fovea VD (%)	32.42 ± 4.62	31.19 ± 4.86	0.041	0.369	−0.365
SCP Parafovea VD (%)	51.40 (49.77–52.02)	51.30 (49.38–51.98)	0.957	0.957	−0.134
SCP Superior VD (%)	51.65 (49.70–53.45)	51.80 (50.02–52.42)	0.859	0.910	−0.013
SCP Inferior VD (%)	50.63 ± 2.49	50.98 ± 1.90	0.456	0.910	−0.129
SCP Temporal VD (%)	51.00 (49.55–52.22)	51.00 (48.90–51.68)	0.513	0.910	−0.221
SCP Nasal VD (%)	50.60 (49.55–52.30)	50.35 (49.40–51.80)	0.768	0.910	−0.185
DCP Parameters					
DCP Whole VD (%)	53.80 (52.20–54.42)	53.80 (52.70–54.08)	0.701	0.910	−0.076
DCP Fovea VD (%)	30.90 (28.73–35.95)	30.30 (27.62–31.60)	0.661	0.910	−0.096
DCP Parafovea VD (%)	54.15 (53.70–54.83)	54.15 (53.52–54.80)	0.844	0.910	−0.194
DCP Superior VD (%)	54.25 ± 1.64	53.82 ± 1.58	0.218	0.654	−0.215
DCP Inferior VD (%)	53.29 ± 2.01	54.37 ± 1.44	0.009	0.162	+0.475
DCP Temporal VD (%)	53.36 ± 2.62	53.91 ± 1.83	0.122	0.549	+0.272
DCP Nasal VD (%)	54.85 ± 2.00	54.16 ± 1.47	0.087	0.522	−0.303
FAZ Parameters					
FAZ Area (mm^2^)	0.205 (0.143–0.278)	0.209 (0.187–0.288)	0.750	0.910	+0.205
FAZ Perimeter (mm)	1.750 (1.580–2.043)	1.798 (1.641–2.075)	0.519	0.910	+0.206
FAZ Circularity	0.820 (0.772–0.840)	0.810 (0.750–0.820)	0.164	0.590	−0.258

Data are presented as mean ± SD for normally distributed variables or median (IQR) for non-normally distributed variables based on Shapiro–Wilk normality testing. Paired *t*-test was used for normally distributed differences, and Wilcoxon signed-rank test was used for non-normally distributed differences. ^†^ Benjamini–Hochberg false discovery rate correction was applied to the exploratory secondary outcomes; the primary outcome (CVI ratio) was pre-specified and excluded from correction. No exploratory parameter remained statistically significant after FDR correction. AL, axial length; BH, Benjamini–Hochberg; CVI, choroidal vascularity index; DCP, deep capillary plexus; FAZ, foveal avascular zone; FDR, false discovery rate; IQR, interquartile range; SCP, superficial capillary plexus; SD, standard deviation; SFCT, subfoveal choroidal thickness; VD, vessel density.

**Table 3:** Spearman Correlation Between Interocular Differences and Head Tilt Degree/Age.

	Head Tilt Degree		Age	
Parameter	ρ	*p* Value	Ρ	*p* Value
**CVI and Choroid Parameters**				
CVI Ratio	+0.533	0.001	−0.046	0.795
SFCT (µm)	+0.302	0.083	−0.009	0.961
**SCP Parameters**				
SCP Whole VD (%)	−0.023	0.896	+0.049	0.782
SCP Fovea VD (%)	−0.017	0.926	−0.008	0.964
SCP Parafovea VD (%)	+0.070	0.694	+0.011	0.949
SCP Superior VD (%)	+0.018	0.922	−0.096	0.589
SCP Inferior VD (%)	+0.144	0.416	+0.087	0.623
SCP Temporal VD (%)	−0.076	0.668	−0.133	0.454
SCP Nasal VD (%)	−0.161	0.363	+0.047	0.791
**DCP Parameters**				
DCP Whole VD (%)	+0.116	0.513	−0.193	0.275
DCP Fovea VD (%)	−0.320	0.065	+0.167	0.346
DCP Parafovea VD (%)	+0.193	0.274	−0.376	0.028
DCP Superior VD (%)	−0.321	0.064	−0.054	0.762
DCP Inferior VD (%)	+0.150	0.396	−0.264	0.132
DCP Temporal VD (%)	+0.019	0.915	−0.206	0.243
DCP Nasal VD (%)	−0.263	0.132	−0.229	0.192
**FAZ Parameters**				
FAZ Area (mm^2^)	−0.306	0.078	+0.356	0.039
FAZ Perimeter (mm)	−0.283	0.105	+0.304	0.080
FAZ Circularity	+0.237	0.176	+0.217	0.219

Δ = Opposite Side − Head Tilt Side. All correlations were performed using Spearman rank correlation. ρ, Spearman correlation coefficient. Positive ρ (Head Tilt Degree) indicates greater opposite-side excess with increasing tilt degree. CVI, choroidal vascularity index; SFCT, subfoveal choroidal thickness; SCP, superficial capillary plexus; DCP, deep capillary plexus; VD, vessel density; FAZ, foveal avascular zone.

## Data Availability

The datasets generated and/or analyzed during the current study are available from the corresponding author on reasonable request.
